# Supplementary material for: Male Stressed Mice Having Behavioral Control Exhibit Escalations in Dorsal Dentate Adult-Born Neurons and Spatial Memory
Source: Int J Mol Sci. 2023 Jan 19;24(3):1983. doi: 10.3390/ijms24031983 (PMC9916676; doi:10.3390/ijms24031983)
Supplement: Supplementary file 1 [file ijms-24-01983-s001.zip › ijms-2126110-supplementary.pdf]

Supplementary Material

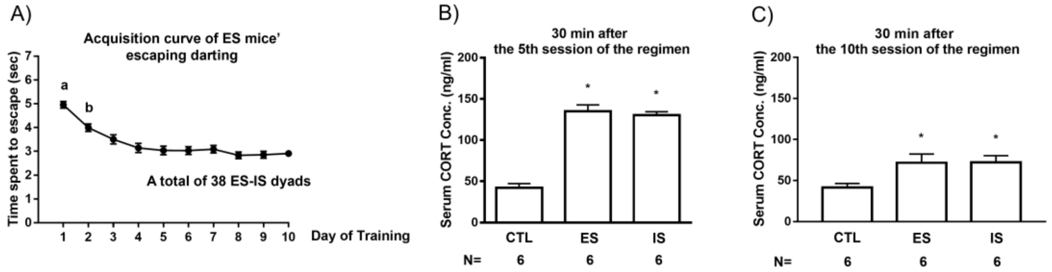

Supplementary Figure S1. Acquisition curve of footshock-terminating darting and footshock regimen-elicited corticosterone (CORT) secretion. A)ES mice seemed to spend comparable durations of darting onset at days 3-10 of the regimen. [a]Significantly higher than the remaining 9 days. [b]Significantly higher than days 3-10. B)ES mice had comparable serum CORT levels as their respective yoke, IS, mice, while both greater than controls at day 5 of the stressor regimen. *Significantly greater than controls. C)ES mice had comparable serum CORT levels as their respective IS mice, while both greater than controls at day 10 of the stressor regimen. *Significantly greater than controls.

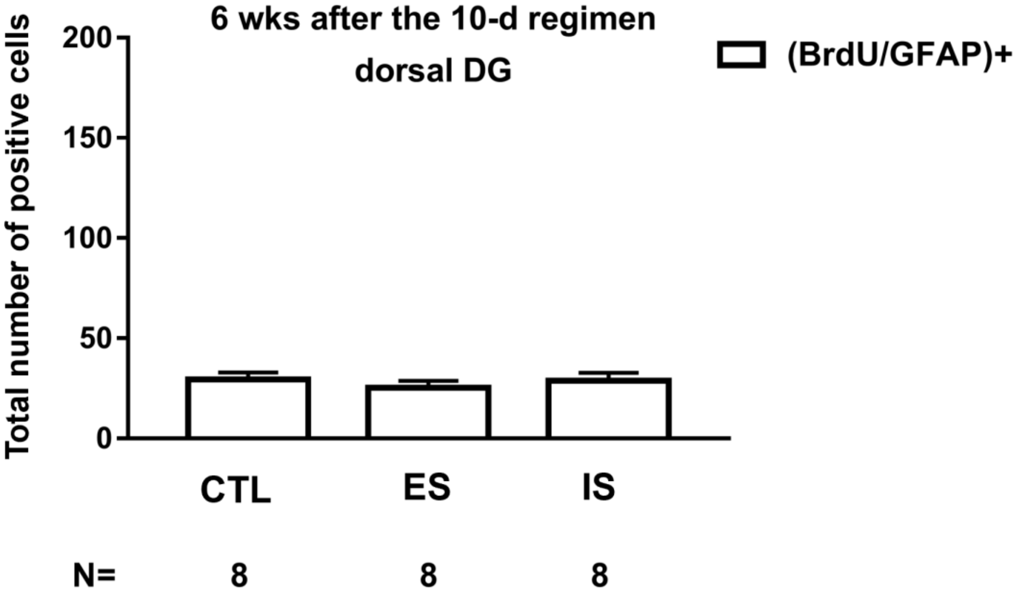

Supplementary Figure S2. Six-weeks-old, BrdU-labelled, mature glial cells in dorsal DG in ES-IS dyads and their respective controls. Three groups of mice had sparse but comparable numbers of BrdU-labelled mature (GFAP-positive) cells in dorsal DG at 6 weeks after the conclusion of the 10-day footshock stressor regimen.

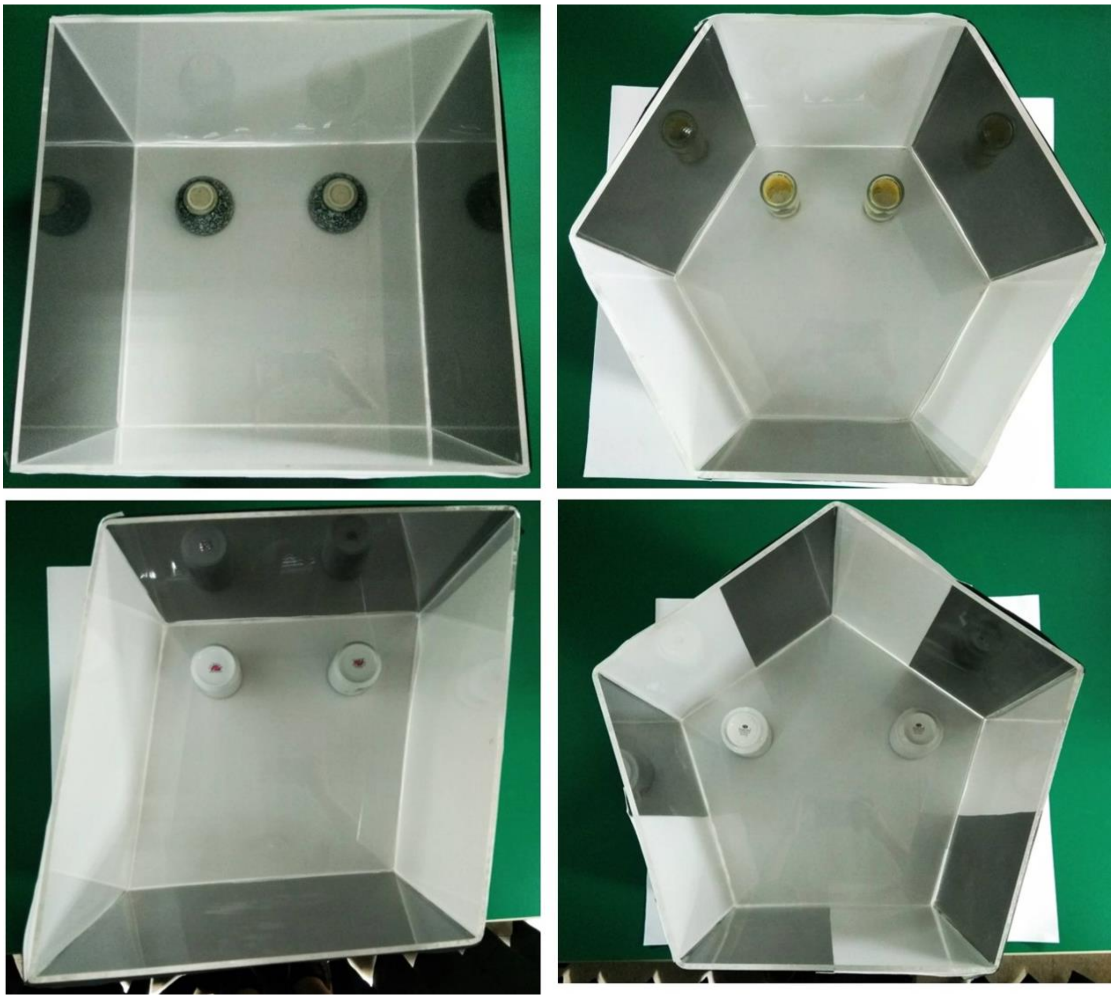

Supplementary Figure S3. Four versions of object location task. (Top left) A rectangle (40 cm x 41 cm) arena (chamber), ceramic cup objects with a bottom diameter of 8.5 cm. (Top right) A regular hexagon arena (chamber) with a side length of 20 cm, ceramic cup objects with a diameter of 4.0 cm. (Bottom left). A parallelogram arena (chamber) with a 36-cm side, ceramic cup objects with a diameter of 6.3 cm. Bottom right) A regular pentagon arena (chamber) with a side length of 27 cm, ceramic cup objects with a diameter of 6.2 cm.

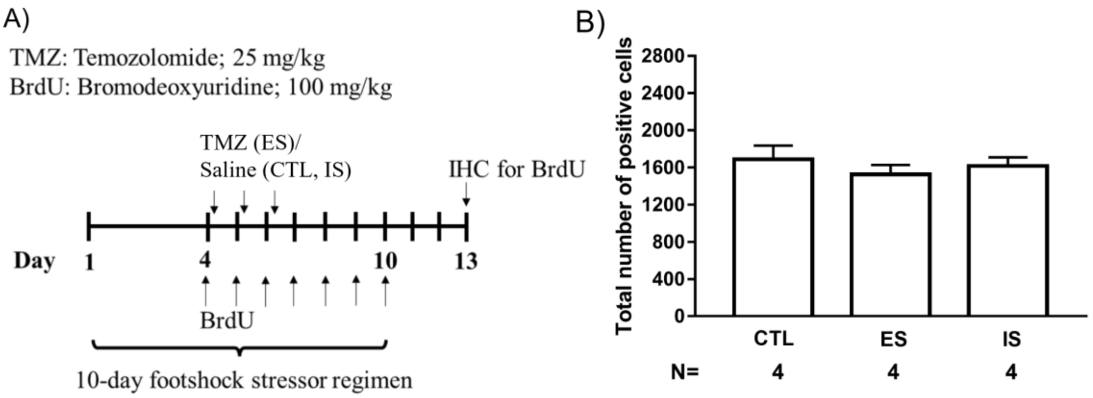

Supplementary Figure S4. Impact of a temozolomide (TMZ) treatment protocol on BrdU-labelled mitotic cells in dorsal DG. A)A timeline for TMZ, BrdU injection protocols and the 10-day footshock stressor regimen. B)Three groups of mice had indistinctive numbers of BrdU-labelled cells in dorsal DG.

Table S1. Experimental purpose and antibodies used

Purpose	Antibody
Short-term effects of repeated stressors and behavioral control on DG neurogenesis	Mouse anti-BrdU<br>Rabbit anti-DCX<br>Alexa Fluor 488-conjugated AffiniPure sheep anti-mouse IgG<br>Alexa Fluor 594-conjugated AffiniPure goat anti-rabbit IgG
Short-term effects of operant conditioning on dorsal DG cell proliferation and early neurogenesis	
Short-term effects of forced running on dorsal DG cell proliferation and early neurogenesis	
Maturation and functional incorporation of the BrdU- labeled cells in dorsal DG at 6 weeks after the conclusion of the 10-day footshock stressor regimen	Mouse anti-BrdU<br>Chicken anti-NeuN<br>Rabbit anti-Arc<br>Rabbit anti-GFAP<br>Alexa Fluor 488-conjugated AffiniPure sheep anti-mouse IgG<br>Alexa Fluor 594-conjugated AffiniPure goat anti-rabbit IgG<br>CF350 conjugated donkey anti-chicken
Long-term effects of the 10-day footshock stressor regimen and behavioral control on dorsal DG mitosis, neuronal density and autophagic flux	**Immunohistochemistry:**<br>Rabbit anti-Ki-67<br>Rabbit anti-NeuN<br>Alexa Fluor 488-conjugated AffiniPure goat anti-rabbit IgG<br>Alexa Fluor 594-conjugated AffiniPure goat anti-rabbit IgG<br>**Western immunoblotting:**<br>Rabbit polyclonal anti-LC3A/B<br>Mouse monoclonal anti-p62<br>Mouse monoclonal anti-β actin<br>Peroxidase-AffiniPure Goat Anti-Rabbit IgG (H+L)<br>Peroxidase-AffiniPure Sheep Anti-Mouse IgG (H+L)
Effects of TMZ treatment on maturation of the BrdU-labeled cells in dorsal DG at 6 weeks after the conclusion of the 10-day stressor regimen	Mouse anti-BrdU<br>Rabbit anti-NeuN<br>Alexa Fluor 488-conjugated AffiniPure sheep anti-mouse IgG<br>Alexa Fluor 594-conjugated AffiniPure goat anti-rabbit IgG
